# Hydrophobic carbon dots with blue dispersed emission and red aggregation-induced emission

**DOI:** 10.1038/s41467-019-09830-6

**Published:** 2019-04-17

**Authors:** Haiyao Yang, Yingliang Liu, Zhouyi Guo, Bingfu Lei, Jianle Zhuang, Xuejie Zhang, Zhiming Liu, Chaofan Hu

**Affiliations:** 1 Guangdong Provincial Engineering Technology Research Center for Optical Agriculture, College of Materials and Energy, South China Agricultural University, 510642 Guangzhou, China; 2 MOE Key Laboratory of Laser Life Science & SATCM Third Grade Laboratory of Chinese Medicine and Photonics Technology, College of Biophotonics, South China Normal University, 510631 Guangzhou, China

**Keywords:** Optical materials, Quantum dots

## Abstract

Carbon dots (CDs) have been studied for years as one of the most promising fluorescent nanomaterials. However, CDs with red or solid-state fluorescence are rarely reported. Herein, through a one-pot solvothermal treatment, hydrophobic CDs (H-CDs) with blue dispersed emission and red aggregation-induced emission are obtained. When water is introduced, the hydrophobic interaction leads to aggregation of the H-CDs. The formation of H-CD clusters induces the turning off of the blue emission, as the carbonized cores suffer from π-π stacking interactions, and the turning on of the red fluorescence, due to restriction of the surfaces’ intramolecular rotation around disulfide bonds, which conforms to the aggregation-induced-emission phenomenon. This on-off fluorescence of the H-CDs is reversible when the H-CD powder is completely dissolved. Moreover, the H-CD solution dispersed in filter paper is nearly colorless. Finally, we develop a reversible two switch-mode luminescence ink for advanced anti-counterfeiting and dual-encryption.

## Abstract (web summary)

Carbon dots that display long-wavelength and multicolor emission are desirable for biological and anti-counterfeiting applications. Here, the authors design hydrophobic carbon dots with reversible two-mode fluorescence, which exhibit blue emission when dissolved in solution, and red solid-state fluorescence when aggregated.

## Introduction

Many types of carbon dots (CDs) have been reported because they are more eco-friendly and potentially a carbon-based fluorescent nanomaterial; however, in solution, most CDs show emission in the blue to green-light regions only^1–3^. Therefore, there is an urgency to attain long-wavelength and multicolor emission of CDs for further applications, particularly in biologically relevant and anticounterfeiting fields^1,2,4–9^. The most common method for inducing long-wavelength and multicolor emission is doping heteroatoms into the lattices of carbon. The addition of heteroatoms leads to defects with the framework of the CDs. Therefore, contraction of the CDs’ band gaps further induces a redshift of the CDs’ fluorescence. Recently, Liu and coworkers fabricated red emission B, N, S-codoped CDs using 2,5-Diaminobenzenesulfonic acid and 4-aminophenylboronic acid hydrochloride. Earlier, Ge et al.^9^ designed a series of Suzuki reactions to synthesize polythiophene derivatives as precursors for red emission S-doped CDs.

However, previous studies on red emission CDs did not obtain CDs that exhibit red fluorescence in the solid state. Most reported CDs only fluoresce when dissolved in solution. The currently accepted mechanism of this phenomenon is similar to the H-aggregation of organic fluorescent molecules; CDs suffer from π–π stacking in the solid state and the aggregation of large conjugated systems consumes the transition energy, therefore, resulting in the quenching of the CDs’ fluorescence. While the extinction of the CDs’ luminescent solid-state has hindered their application in LED and anticounterfeiting technology^8,10–13^. The current method to maintain CDs’ solid-state-fluorescence (SSF) is to block the CD monomers from direct contact. Most reported studies on SSF CDs attempt to dope CDs into matrices or introduce polymer chains into CDs^8,11^. For instance, Chen et al.^13^ prepared N-doped CD’s (NCDs) with yellow-green SSF by hydrothermal treatment of poly (vinyl alcohol) (PVA) and ethylenediamine (EDA). The abundant surface PVA chains covered around the NCDs prevented the graphitizing cores from π−π interactions; thus, resisting the aggregation-caused-quenching (ACQ) of the NCDs’ fluorescence. However, these studies did not achieve red SSF of CDs. Moreover, the introduction of matrices or polymer chains restricted the concentration of the doped CDs; if too many CDs are introduced, their ACQ will still take place.

Unlike the ACQ^14^ property of nanomaterials, B.Z Tang^15–18^ and coworkers discovered a series of organic fluorescent materials and found that luminogen aggregation played a destructive role in the light-emitting process. In their studies, a series of symmetrical molecules were found to be nonluminescent in the dissolved state, but emissive in the aggregated state. The term “aggregation-induced emission” (AIE) was coined for this phenomenon, as the nonluminescent symmetrical molecules were induced to emit via aggregate formation. This theory has not been utilized in the SSF of carbon dots yet.

Incidentally, we find another approach for maintaining a CDs’ SSF, in addition to introducing them into solid dispersed systems: crowning the CDs’ graphitized cores with rotatable symmetric surfaces, through a series of amidation and rearrangement during a solvothermal carbonization process. When fully dispersed as a homogeneous solution, our CDs exhibit similar PL characteristics as the reported blue emission CDs. By adding water, the CDs continuously assemble due to their hydrophobicity, the blue fluorescence turns off while a red SSF turns on. Like AIE molecules in solution, the surficial groups of CDs can rotate around the intramolecular disulfide bonds and consume the absorbed energy, thus, not producing fluorescence. However, in the solid state, as a result of the intramolecular rotation being banned, the excitation energy can transfer dominantly into fluorescence. Therefore, we have designed a method to synthesize hydrophobic N, S-doped CDs (H-CDs) with a two-switch-mode luminescence between a blue dissolved fluorescence and a red AIE. Moreover, the output of the H-CD powders is higher (after purification, the mass ratio of H-CD powders to raw materials is approximately 80%) than prior methods. Several characterizations are taken to determine the properties of H-CDs. When H-CD powder dissolves into certain organic solvents (ethanol or acetic acid), it displays the same blue fluorescence as the as-prepared H-CD solution. However, in DMF the H-CD displays both blue and red fluorescence, due to the existence of mono-dispersed and aggregated H-CDs. To confirm the fluorescence mechanism and the relationship between the H-CD dispersed state and fluorescence, we have designed a control experiment (replaced dithiosalicylic acid with benzoate to remove the disulfide bonds). Finally, we fill the as-prepared H-CD solution into a mark pen and conduct a series of anticounterfeit and encryption experiments to develop a reversible two-switch-mode luminescence ink.

## Results

### Preparation and characterization of the H-CDs

**Fig. 1:**
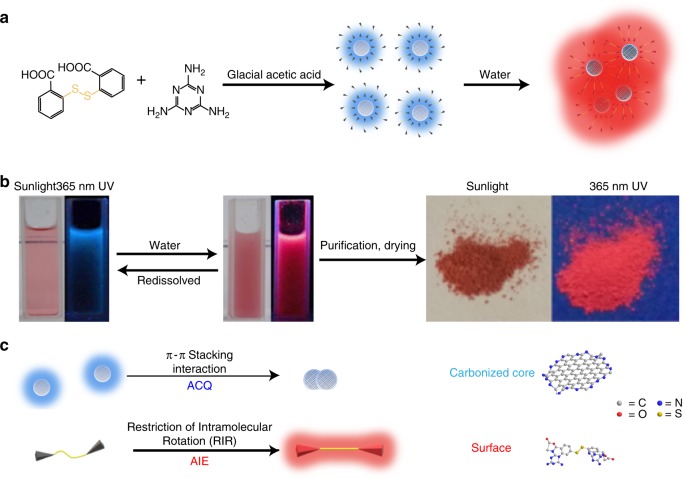
H-CDs’ formation, structure, fluorescence and appearance in different state. **a** Formation of H-CD monomers and their aggregates (the disulfide bond in dithiosalicylic acid molecular is highlighted with yellow). **b** Photographs of the H-CDs’ two-switch-mode luminescence principle. **c** Fluorescence principle and proposed structure of H-CD’s core and surface (the colors of glowing edges represent the color of their fluorescence). H-CD, hydrophobic carbon dot

The H-CD powder was easily prepared through a one-pot solvothermal process of melamine (MA) and a dithiosalicylic acid (DTSA)/acetic acid solution, followed by a simple purification (Fig. 1). It should be noted that acetic acid plays a vital role during the formation of H-CDs. In addition to being an environmentally friendly solvent with low cost, it is also a catalyst for H-CDs’ carbonization and the constitution of H-CD surface (Supplementary Fig. 1a). To further investigate the effect of acetic acid, we applied a series of control experiments which replaced acetic acid with formic acid, propionic acid and saturated aqueous solution of oxalic acid. When propionic acid is added, the product (named as PA-CDs) shows a similar PL property as the H-CDs: blue emission in dispersion and yellow AIE in the powder state (Supplementary Fig. 1b, c). However, propionic acid is much more expensive and toxic than acetic acid, and the fluorescence of PA-CD powder is yellow unlike the red AIE of the H-CDs. Through continuous water addition, the transparent as-prepared H-CD solution gradually turns into a turbid liquid, and the blue emission fades away. Then, a red fluorescence emerges. H-CD powder displaying red SSF under 365 nm UV irradiation can be obtained with further purification and drying. Remarkably, under 2 nm UV, the red emission of the H-CD powder remains while the H-CD dispersion displays nearly no fluorescence.

**Fig. 2:**
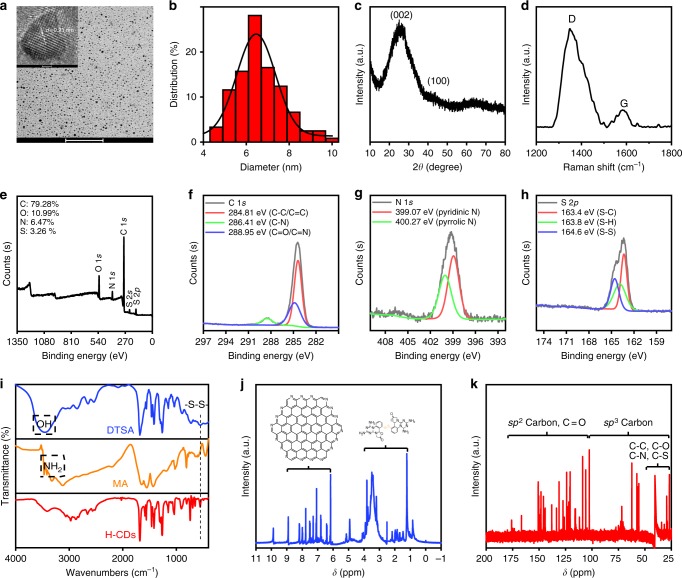
Basic characterizations of H-CDs. **a** TEM image of the H-CDs, inset: high-resolution TEM (HR-TEM) image of the H-CDs. **b** Particles size distribution measured by TEM. **c** X-ray diffraction (XRD) pattern of the H-CDs. **d** Raman spectrum of the H-CDs. **e** XPS spectrum and high-resolution **f** C 1*s*, **g** N 1*s*, and **h** S 2*p* spectra of the H-CDs. **i** FT-IR spectrum of DTSA, MA and the H-CDs (the position marked by dotted rectangles refer to hydroxyl and amino group, peaks belong to disulfide bond are marked by dotted line). **j** ^1^H NMR (insets: proposed structure of the H-CD core and surface, the braces mark out the regions they belong to separately) and **k** ^13^C NMR spectra (the braces mark out the regions related to carbon different with different molecular orbital) of the H-CDs in DMSO-d6. Scale bars: 100 nm (**a**) and 10 nm (**a**—inset). H-CD, hydrophobic carbon dot, TEM transmission electron microscopy

The as-prepared H-CDs have been characterized with transmission electron microscopy (TEM), X-ray diffraction (XRD) and Raman spectroscopy to confirm the nature of the carbon nanoparticles. As shown in Fig. 2a, b, the TEM image of the H-CDs presents size distributions between 4 and 10 nm, with an average diameter of approximately 6.5 nm. High-resolution TEM (HR-TEM) shows a lattice spacing of 0.21 nm corresponding to the (100) facet of graphite and reveals that the H-CDs contain graphite-like structures^19–21^. The XRD pattern of the H-CDs (Fig. 2c) has an apparent peak at approximately 25°, which is attributed to an interlayer spacing of 0.34 nm, while the peak near 41° represents the 0.21 nm interlayer spacing^5,8,13,22^. The Raman spectrum in Fig. 2d displays two peaks at 1348 cm^−1^ (D band) and 1584 cm^−1^ (G band), referring to areas of disordered surfaces and *sp*^2^ carbon networks in the H-CDs’ frameworks, respectively. The calculated intensity ratio *I*_D_/*I*_G_ is 5.61, indicating the amorphous surface of the H-CDs^1,5,6,19^.

Fourier transform infrared (FT-IR) spectra, X-ray photoelectron spectra (XPS) and nuclear magnetic resonance (NMR) spectroscopy were taken to further analyze the chemical structure of the H-CDs. The FT-IR spectrum (Fig. 2i) uncovers that the surface of the H-CDs contains methylene (2876 and 2973 cm^−1^), C≡N (2034 cm^−1^), S−H (2650 cm^−1^), amide carbonyl (1682 cm^−1^), C=C (1469 cm^−1^), C−N (1407 cm^−1^), C−S (685 cm^−1^), S−S (491 cm^−1^), aromatic C−NH (1261 cm^−1^) and C−O (1124 cm^−1^) functional groups or chemical bonds. Additionally, the FT-IR spectra of MA and DTSA exhibit that these raw materials contain an hydroxyl or amino (3064 and 3411 cm^−1^)^1,4,8^. Furthermore, after the amidation and carbonization, these hydrophilic groups almost disappear in the H-CDs, thus, contributing to the hydrophobic properties of the H-CDs^23–34^. The full XPS spectrum presented in Fig. 2e shows four peaks at 284.81, 399.62, 532.22, and 163.89 eV, suggesting that the H-CDs consisted of C, N, O, and S elements, and the atomic ratios were calculated to 79.28%, 6.47%, 10.99%, and 3.26%, respectively. In Fig. 2f, the high-resolution XPS spectrum of the C 1*s* band was separated into three peaks at 284.81, 286.41, and 288.95 eV, which are assigned to C–C/C=C, C–N and C=O/C=N, respectively. The N 1*s* band (Fig. 2g) exhibits two peaks at 399.07 and 400.27 eV, respectively, which correspond to pyridinic C_3_–N and pyrrolic C_2_–N–H groups. The S 2*p* band in Fig. 2h contains three peaks at 163.35 eV for S−C, 163.81 eV for S–H and 164.57 eV for S–S. These three high-resolution spectra collectively indicate the successful insertion of S and N atoms into the H-CDs. Furthermore, NMR spectra (^1^H and ^13^C) were employed to distinguish the *sp*^3^-hybridized carbon atoms from the *sp*^2^-hybridized carbon atoms (Fig. 2j, k). Deuterium-labeled DMSO-d6 (CD_3_SOCD_3_) was used as a solvent. In the ^1^H NMR spectra, *sp*^2^ carbons were detected. The peak at 9.99 ppm in Fig. 2j is the chemical shift of the carboxyl protons. Furthermore, signals from the aromatic rings are detected at 8.3 ppm, which can be attributed to graphitized cores’ proton resonances. The emergence of the –NH_2_ protons at 5.75 ppm implies the introduction of primary amines into the heterocyclic surface^19,22,35^. In the ^13^C NMR spectrum, signals in the range of 30−45 ppm are associated with the aliphatic (*sp*^3^) carbon atoms, and signals from 100 to 185 ppm are indicative of *sp*^2^ carbon atoms. Signals in the range of 170−185 ppm correspond to carboxyl/amide groups^36–38^. Based on the aforementioned characterizations, which support the reaction mechanism proposed in Supplementary Fig. 1a, a molecular model for the H-CDs can be constructed: a nanoscale graphite-like skeleton with defects caused by pyridinic nitrogen atoms and disulfide bonds, covered with C, N, O and S containing symmetrical heterocycle rotatable structures. Notably, there are few amino and hydroxyl functional groups on the surface of the H-CDs, which is quite different from the water-soluble CDs reported in prior works. This model explains the hydrophobicity and optical properties.

### Optical properties and fluorescence mechanism of the H-CDs

**Fig. 3:**
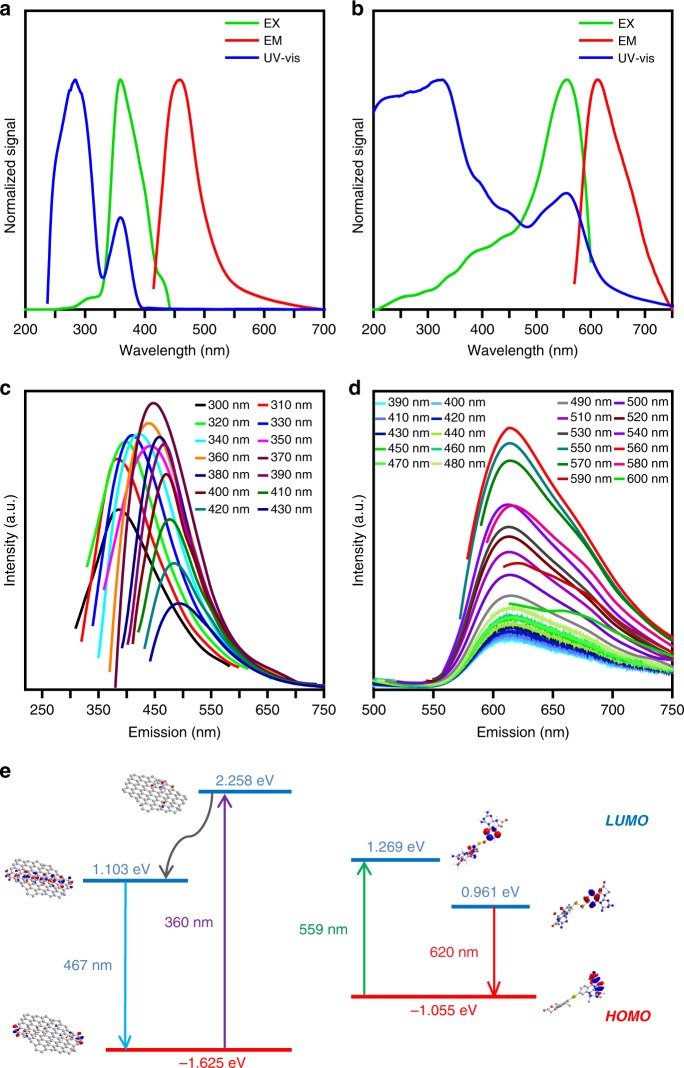
Absorption and emission properties of H-CDs and concerned orbital energy level. **a** UV−Vis absorption (blue line), PL excitation (EX) (*λ*_em_ = 467 nm, orange line), and emission (EM) (*λ*_ex_ = 360 nm, red line) spectra of as-prepared H-CD solution. **b** UV−Vis absorption (green line), PL excitation (Ex) (*λ*_em_ = 620 nm, orange line), and emission (Em) (*λ*_ex_ = 559 nm, red line) spectra of the H-CD powder. **c**, **d** PL emission spectra with different excitation wavelengths of H-CD powder and as-prepared solution. **e** Energy level diagram of proposed H-CDs’ graphitizing core, and the surface symmetrical heterocycle’s molecular orbital. H-CD, hydrophobic carbon dot

The UV−Vis absorption, PL excitation, and emission of the as-prepared H-CD solution and powder were examined to evaluate their optical properties. As shown in Fig. 3a, the UV−Vis absorption of the as-prepared H-CDs has two peaks at *λ*_max1_ ≈ 280 nm and *λ*_max2_ ≈ 360 nm due to the π–π* transitions of the C=C in the core of the H-CD. While the H-CD powder exhibits a different broad absorption, with a dominating band at *λ*_max_ ≈ 560 nm (Fig. 3b), which is attributed to the n–π* transitions of the surface states containing C=N/C=O, C–O and C−S structures. Figure 3d represents the PL emission of the H-CD powder under different excitation wavelengths, showing a stable red emission at *λ*_max_ ≈ 620 nm, with a different excitation wavelength that is more similar to traditional inorganic phosphors than reported CDs. However, the as-prepared H-CD solution (Fig. 3c) exhibits excitation-dependent PL features; similar to most CDs in prior works, the optimal excitation and emission are near 360 and 467 nm^1,6,10,11,20,39^, respectively. The computational process of the H-CDs’ molecular orbital energy level and fluorescence lifetime (4.56 ns) is described in the “Methods” section. The quantum yield of the H-CDs can be calculated as 5.96%, due to their photon absorption and emission (Supplementary Fig. 2b, c)^4,7,11,21,40,41^. Visually, bright field and fluorescence microscopy images (Supplementary Fig. 3) of excess H-CD powder in ethanol indicate that thick stacking H-CD powder produces red fluorescence, and the dissolved H-CD solution displays blue emission. At the thin periphery of the H-CD powder infiltrated with the solution, the red and blue emission hybridize together, giving rise to a pink hybrid fluorescence^8,10,13^.

**Fig. 4:**
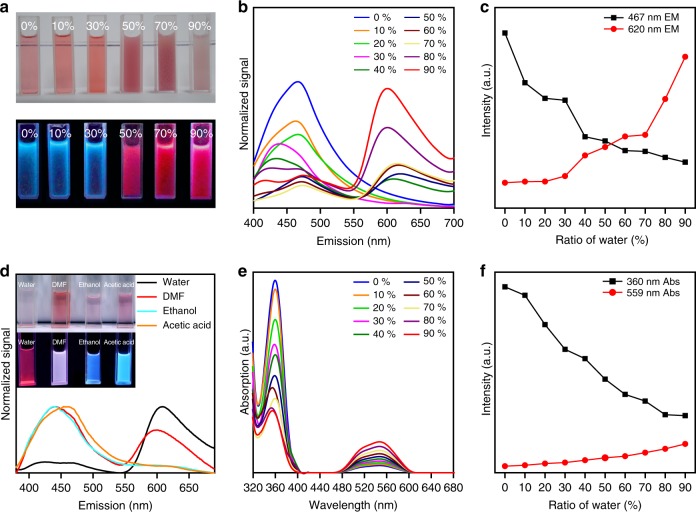
H-CDs’ absorption and emission varied with different solvent. **a** Photographs of the as-prepared H-CD solution with varying volume ratios of water (from 0 to 90%) under sunlight (top) and 365 nm ultraviolet radiation (bottom). **b** PL emission spectra of the H-CD as-prepared solution with varying ratios of water. **c** Trend of the H-CD as-prepared solutions’ fluorescence intensity at 467 and 620 nm, varying with the ratio of water. **d** PL emission spectra of the H-CD powder solutions in different solvents (insets: photographs of the H-CD powder solutions in solvents with different polarities (from high to low) under sunlight (top) and 365 nm ultraviolet radiation (bottom)). **e** UV−Vis absorption spectra of the H-CD as-prepared solution with different ratios of water. **f** Trend of the H-CD as-prepared solutions’ absorbance at 360 and 559 nm, varying with the ratio of water. H-CD, hydrophobic carbon dot

More intuitively, the photographs in Fig. 4a show that the pure as-prepared H-CD solution and the solution with an addition of less than 50% water (volume ratio) are orange to red homogeneous and transparent liquids. When the volume ratio of the water is greater than 50%, the as-prepared H-CD solution starts to separate the red powder out and turns into a turbid liquid with a suspension. Under a 365 nm UV excitation (Fig. 4b), the aforementioned transparent liquids display blue fluorescence, while the turbid liquid with the suspension glows red. In addition, correlated to the variation trend of the size distribution by adding more water, the intensity of the H-CD solution’s blue emission decreases, while the red emission is heightened (Fig. 4c). The H-CDs in solvents with different polarities exhibit a similar fluorescence phenomenon (Fig. 4d) to the as-prepared H-CDs with varying ratios of water. The red emission increases when the polarity of the solvent decreases. The UV−Vis absorption spectra and absorbance trend of the H-CD solutions with varying ratios of water (Fig. 4e, f) reveal that with the injection of water, the absorbance at 360 nm continues decreasing, while a redshifted absorbance at 559 nm appears and continues increasing. This trend provides strong evidence for the presence of π−π stacking in the H-CDs. Conjugated systems can form two distinct types of π−π aggregates, a sandwich-type arrangement (H-aggregates) and a head-to-tail arrangement (J-aggregates)^42^. According to the molecular exciton coupling theory, the spectral redshift indicates that the H-CDs form J-aggregates, with a head-to-tail arrangement^43–46^. Based on the red SSF of the H-CD powders, a fluorescent organic glass was fabricated and assembled into a WLED with a cyan LED-chip (Supplementary Fig. 4).

**Fig. 5:**
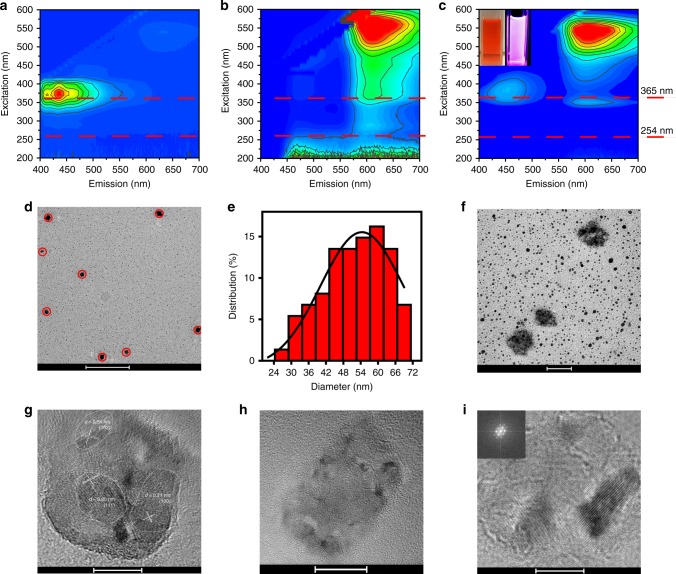
Different fluorescence and morphology of H-CDs in varying state. PL mapping spectra of **a** the H-CD powder acetic acid solution, **b** H-CD powder and **c** the H-CD powder DMF solution (insets: photos of the H-CD DMF solution under white light (left) and 365 nm (right) irradiation, dash line marks out emission under 365 or 254 nm UV). **d**, **f** TEM image and **e** particles size distribution of H-CD aggregates (marked by red circles) in the DMF solution. **g**–**i** High-resolution TEM (HR-TEM) image of the H-CD aggregates in the DMF solution, inset: FFT diffraction pattern of carbon lattice. Scale bars: 500 nm (**d**), 50 nm (**f**), 20 nm (**h**) and 10 nm (**g**, **i**). H-CD, hydrophobic carbon dot, TEM transmission emission microscopy

As mentioned above, when the H-CD powders dissolve into DMF, an orange transparent liquid is obtained (Fig. 5c, left inset). Under 365 nm UV excitation, the H-CD powder DMF solution exhibits a pinkish red fluorescence (Fig. 5c, right inset). The PL mapping spectrum (Fig. 5c) reveals that there are both blue and red emission centers in the H-CD powder DMF solution. Relatively, the H-CD powder acetic acid solution (Fig. 5a) has only a blue emission center, and the H-CD powder (Fig. 5b) solely obtains a red emission center. TEM images (Fig. 5d–f) indicate that the H-CD aggregates, with an average diameter of approximately 56 nm, exist in the DMF solution around the H-CD monomers. The HR-TEM images (Fig. 5g–i) of the H-CD aggregates, and the FFT diffraction pattern (inset of Fig. 5i) of the carbon lattice reveals there are different carbon lattice planes in the H-CD aggregates, meaning that the H-CDs assemble with random orientation^47–49^. Therefore, the H-CD aggregates generate red SSF and the monomers contribute to the blue emission, which reveals the relationship between the H-CDs’ luminous mechanism and their dispersed state.

To further verify the structure and fluorescence mechanism of the H-CDs, we carried out two control experiments, the first displaced DTSA with benzoate to remove the effect of the disulfide bonds. The produced CDs are named B-CDs. As shown in Supplementary Fig. 5a, c, the as-prepared B-CDs solution displays similar blue emission to the H-CDs. However, the B-CDs in the solid state exhibit no fluorescence (Supplementary Fig. 5b, d). Moreover, the solid-state B-CDs can easily dissolve into water (Supplementary Fig. 5e). According to the FT-IR spectra of B-CDs and H-CDs, the chemical structure of the B-CDs is similar to the H-CDs, except for the disulfide bonds. Thus, the relationship between the symmetrical surface around the disulfide bonds and the H-CDs’ hydrophobicity and red AIE can be affirmed.

The second control experiment used a postmodification method to synthesize dithiosalicylic acid-modified CDs, which are named P-CDs. First, MA is dissolved into acetic acid and undergoes a solvothermal pretreatment. The P-CD intermediate is water-soluble and displays blue fluorescence (Supplementary Fig. 6a). The TEM image of this intermediate in Supplementary Fig. 6d and its inset indicates that a carbonized dot structure with a 0.25 nm lattice spacing (111 lattice plane of carbon), which can further verify that the blue emission of the H-CDs comes from its carbonized core. P-CDs were then fabricated by mixing DTSA with the aforementioned intermediate and acetic acid, after a post-solvothermal processing. As shown in Supplementary Fig. 6b, c, e, f, P-CDs exhibit same hydrophobicity and PL properties as the H-CDs, which confirms the root of the H-CDs’ hydrophobicity and red AIE is the DTSA-modified surface.

Therefore, we can build a model comprised of the core formed by MA with an N, S, O-containing, rotatable symmetrical heterocyclic surface. Optical properties and calculated energy level transitions reveal the correspondence of blue emission to the core and red emission to the surface. Photoluminescence videos (Supplementary Movies 1, 2, 3) of the H-CDs in different dispersed-states suggest that H-CDs show blue emission in a dissolved state, and red emission in a solid state. The H-CD ethanol solution was added onto a copper grid and dried, then deionized water was sprayed onto the copper grid. The TEM image (Supplementary Fig. 7) of the copper grid revealed that the H-CD monomers were becoming closer than H-CD solution displayed in Fig. 2a. Thus, a convincible aggregate and luminous mechanism can be proposed: in solution, H-CDs’ graphitized cores are dominant while the rotatable symmetrical heterocycles around the disulfide bond is recessive; therefore, the H-CD solution exhibits excitation-correlated blue fluorescence, similar to reported carbon dots. When the H-CD monomers contact water, the hydrophobicity of their surfaces cause them to approach each other. Then the conjugated system of the surfaces conducts π−π stacking to overlay each other. Finally, the H-CDs take the shape of J-aggregates. Due to this aggregation, the graphitized cores will suffer a π−π stacking interaction and further turn off the blue emission via ACQ. Furthermore, the axisymmetric heterocycles shown in Supplementary Fig. 2 suffer from the restriction of intramolecular rotation (RIR) of symmetrical heterocycles about their disulfide bonds axes, like other reported symmetrical molecules with AIE^17,18,50^, resulting in red AIE^15,16,51^.

### H-CD-based two-switch-mode luminescence ink

**Fig. 6:**
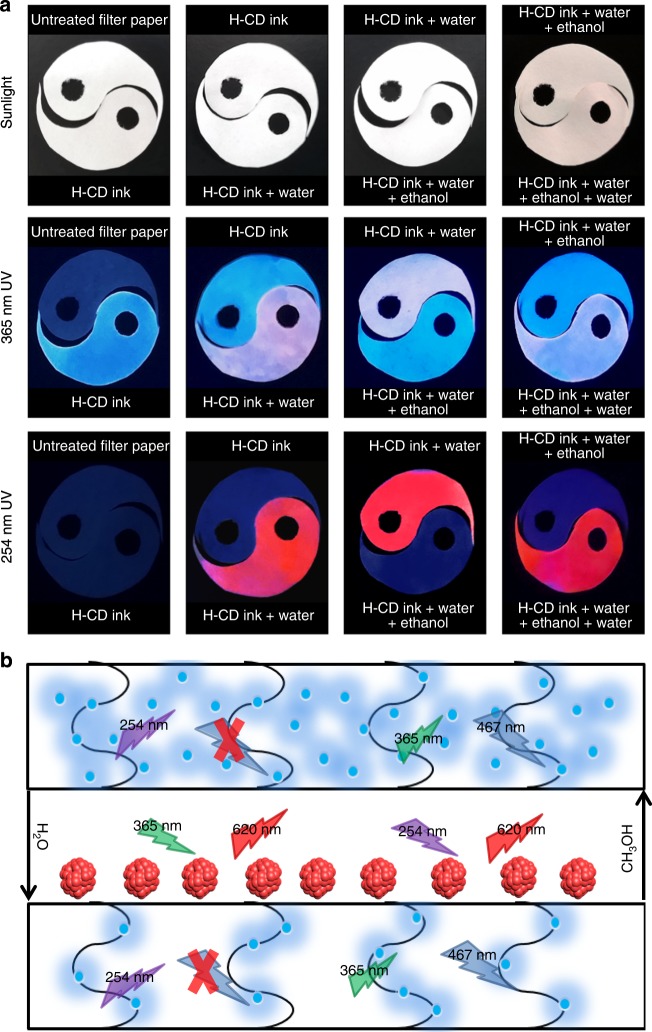
Presentation and principle of H-CDs’ reversible dual-fluorescence. **a** Photographs of filter papers with different treatment under variable irradiation. **b** Schematic diagram of the H-CDs precipitated with water addition and redissolving into ethanol, with the resulting multimode fluorescence. (The square frames represent the filter paper, the wavy lines represent the paper’s fibers, the blue dots represent the H-CD monomers, the flash symbols upward refer to admit light while the downwards ones present emission, the red crosses mean no emission). H-CD, hydrophobic carbon dot

As shown in Fig. 6a, the as-prepared H-CD solution was painted on a filter paper. Under white light, it is almost colorless and shows a blue fluorescence under 365 nm UV excitation. At 254 nm the UV irradiation cannot produce any fluorescence, which conforms to the PL property of the H-CD monomers shown in Fig. 5. By adding water and air-drying, its fluorescence under 365 nm UV turns to pink. Furthermore, it appears as a red fluorescence, which suggests the H-CDs on the filter paper contains both H-CD monomers and H-CD aggregates, compared with the former data. With the addition of ethanol and air-drying, the liquid H-CD displays the same optical properties as the H-CD monomers. Furthermore, the addition of water can turn on the red emission again. This phenomenon suggests that the as-prepared H-CD solution can be utilized as a reversible two-switch-mode ink. A schematic mechanism for the ink is shown in Fig. 6b. The square frames in Fig. 6b represent the filter paper, the wavy lines represent the paper’s fibers. Blue dots represent the H-CD monomers dispersed in the filter paper due to the restriction of the paper’s fibers. As mentioned above, H-CD monomers cannot be excited at 254 nm but can be excited at 365 nm. When water is introduced, some of the H-CD monomers aggregate and surface. Furthermore, the other monomers remain joint to the fibers. Therefore, under 365 nm irradiation, both the monomers in the filter paper and the aggregates on surface can be excited to glow blue and red emission, which display as a hybrid pink fluorescence. While under 254 nm irradiation, the monomers are not excited further, resulting in the red emission only. Once ethanol is applied, the aggregates will dissolve into the filter paper as monomers again; therefore, this process is reversible. A video has been taken to show this reversible process (Supplementary Movie 4). In this video, we observed that the transfer of the different fluorescence is extremely fast. The excellent reversibility of the process makes the H-CDs promising candidates for practical anticounterfeiting and encryption applications.

**Fig. 7:**
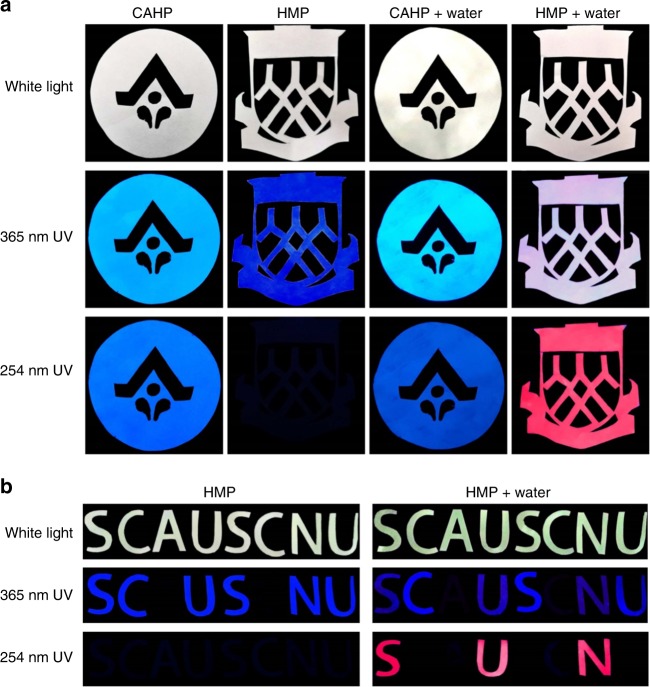
Application of H-CDs ink. **a** H-CD as-prepared solution-filled mark pen (HMP) utilized as an anticounterfeiting badge compared with commercially available highlighter pen (CAHP); **b** HMP utilized as a dual-encryption badge. H-CD, hydrophobic carbon dot

Finally, the as-prepared H-CD solution was filled into an empty mark pen to form a convenient anticounterfeiting and encryption tool. Two school badges painted with a commercially available highlighter pen (CAHP) and an H-CD as-prepared solution-filled mark pen (HMP) (Fig. 7a) based on the filter papers were fabricated. The badges underwent the same treatments as Fig. 6a, c in order. Under white light, the badges are as white as empty filter papers. The CAHP-painted badge exhibits cyan fluorescence under 365 nm UV and blue emission under 254 nm UV. Additionally, water addition does not make an obvious change. While under the different treatments and irradiation, the HMP-painted badge can display four different luminescence characteristics (with HMP, under 365 nm UV, blue emission; under 254 nm UV, no emission; with HMP and water, under 365 nm UV, pink emission; under 254 nm UV, red emission). Evidently, the H-CD as-prepared solution-filled mark pen manifests distinctly unique luminescent properties and stability through the injection of different solvents. The HMP dual-encryption utilization is presented in Fig. 7b. “SC”, “US” and “NU” are painted by HMP; moreover, “C”, “S” and “U” are covered with wax after the ink is air-dried. With 365 nm UV excitation, with or without water, only a series of meaningless fake-codes are shown in blue fluorescence. With 254 nm UV irradiation and no water addition, only darkness is observed. Specifically, the true code “SUN” appears as red fluorescence with simultaneous water treatment and 254 nm UV excitation.

## Discussion

In summary, H-CDs with reversible two-switch-mode luminescence (blue dissolved fluorescence and red AIE) are attained from an eco-friendly, low-cost one-pot solvothermal treatment. TEM, XRD and Raman spectra were taken to confirm the nature of the H-CD carbon nanoparticles. Afterwards, the hydrophobic disulfide bond, containing a symmetrical heterocyclic surface of H-CDs was confirmed by XPS, FT-IR spectra and NMR. The hydrophobicity stems from the abundance of pyridinic and epoxy groups on their symmetrical heterocyclic surfaces. As dispersed monomers in organic solvents, such as AC and ethanol, the as-prepared CDs display a “traditional” ultraviolet absorption (*λ*_EX_ = 315 nm) and blue emission (*λ*_EM_ = 467 nm). The as-prepared CDs are extremely hydrophobic because of the epoxy and pyridyl groups on their surfaces. Thus, the as-prepared CDs will precipitate if water is injected. The precipitations show no blue emission, as the CDs aggregate into J-aggregates and the carbonized cores suffer from aggregation-caused-quenching (ACQ) due to the π−π stacking interaction of their vast conjugated system. Furthermore, the dominated surficial energy transition turns into the production of fluorescence, as the intramolecular rotation of the symmetrical heterocycles about the disulfide bonds is restricted, leading to red AIE (*λ*_EM_ = 621 nm, *λ*_EX_ = 559 nm). This mechanism is confirmed by the aforementioned characterization methods as well as experiments on monomers and aggregates of H-CDs in a DMF solution: pure H-CD monomers in AC or ethanol solutions solely exhibit blue emission; pure H-CD powder only show red AIE; in a DMF solution, when the monomers and aggregates of H-CD coexist, the blue dissolved fluorescence and red AIE take place. Two control experiments were conducted to further confirm this theory. The turning off of the H-CDs’ blue emission and turning on of the red AIE is reversible. As a result, an anticounterfeiting fluorescence ink for advanced anticounterfeiting and dual encryption has been fabricated based on the two-switch-mode luminescence of H-CDs.

## Methods

### Materials

Melamine, dithiosalicylic acid, and benzoate were obtained from Shanghai Adamas Reagent Co., Ltd. Acetic acid was purchased from Guangdong Guanghua Sci-Tech Co., Ltd. All reagents were of analytical grade and used directly without further purification. Deionized water was produced through a Millipore water purification system (Milli-Q, Millipore) and used throughout the study.

### Instruments and measurements

UV−Vis absorption spectra of the powder samples were performed using a Shimadzu UV-2550 ultraviolet-visible spectrophotometer. PL spectra were measured using a Hitachi FL7000 fluorescence spectrophotometer instrument apparatus. The XRD pattern was collected using a XD-2×/M4600. The HR-TEM images were recorded using a JEOL-2010 electron microscope. FT-IR spectra were taken on a Nicolet Avatar 360 FT-IR spectrophotometer. X-ray photoelectron spectroscopy (XPS) experiments were performed using a Kratos AXIS Ultra DLD X-ray photoelectron spectrometer with a monochromatic Al Kα X-ray source. Raman spectra were obtained by a Renishaw via a microspectrometer with an excitation wavelength of 785 nm laser. Particle size analysis is achieved from a Malvern Nano 2SE laser particle size analyzer. NMR measurements were taken by AVANCEIII500 (Bruker). The absolute quantum yield and lifetime are respectively measured by a Hamamatsu C11347 and a Quantaurus Tau C11367.

### Synthesis of the H-CDs

201.6 mg MA and 544 mg DTSA were dissolved into 40 mL acetic acid with ultrasonic treatment, then the solution was transferred into an 80 mL Teflon reactor and kept at 180 °C for 10 h in an air oven. After the solvothermal treatment, the as-prepared H-CD solution was added into 1 L boiled water to form H-CD powder and wash out the residual raw materials and solvent. Finally, purified H-CD powder was achieved through vacuum filtration. To confirm the reliability of this water-wash method, we applied column chromatography to purify the H-CD solution for comparison, and the H-CDs obtained from this approach are named CC-H-CDs. FT-IR and UV−Vis spectra of H-CDs and CC-H-CD powders in Supplementary Fig. 8 suggest the components are approximately identical. Therefore, the water-wash method is considered as reliable as column chromatography.

### Synthesis of the P-CDs

201.6 mg MA was dissolved into 40 mL acetic acid with ultrasonic treatment, then the solution was transferred into an 80 mL Teflon reactor and maintained at 180 °C for 5 h in an air oven. The as-prepared solution was purified by centrifuge and column chromatography and intermediate powders were collected from freeze-drying. Afterwards, 150 mg intermediate powder and 544 mg DTSA were dissolved into 40 mL acetic acid, transferred into an 80 mL Teflon reactor, and maintained at 180 °C for 5 h in an air oven. After the solvothermal treatment, the as-prepared H-CD solution was added into 1 L boiled water to form H-CD powders and wash out residual raw materials. Finally, the purified P-CDs powder was obtained through vacuum filtration.

### Synthesis of the B-CDs

201.6 mg MA and 434 mg were dissolved into 40 mL acetic acid with ultrasonic treatment, then transferred into an 80 mL Teflon reactor and maintained at 180 °C for 10 h in an air oven. After the solvothermal treatment, the as-prepared B-CDs solution was dialyzed in deionized water for a week to remove residual raw materials. Finally, the purified solid-state B-CDs were obtained through freeze-drying.

### Preparation of H-CD-powders-based fluorescence organic glass

150 mg H-CD powders, 53.7 mg dibenzoyl peroxide, 1 mL dibutyl phthalate and 15 mL methyl methacrylate were added into a 250 mL flask, the mixture was maintained at 90−92 °C for 15 min in water bath. Afterwards, the flask was cooled down to 40 °C rapidly, and the mixture was poured into a template and maintained at 100 °C for 2 h. After reverse molding, fluorescent organic glasses (Supplementary Fig. 4) based on H-CD powders were obtained.

### Computational process of H-CDs’ molecular orbital energy level and fluorescence lifetime

The energy level transitions (Fig. 3e) of the H-CDs’ carbonized cores and symmetrical heterocyclic surfaces were calculated by the formula

1 $${E} = h\frac{c}{\lambda }$$

according to their absorptive and emissive properties, which fit the molecular orbital energy level of the proposed structure simulated by the Gaussian 09 plug-in in ChemBioOffice 2014®. The fluorescence decay curve and double-exponential fitting results are shown in Supplementary Fig. 2a. The fitting function is shown in the formula below

2 $${{y}} = {{y}}_0 + A_1 \ast {\mathrm{exp}}( - (x - x_0)/{{t}}_1) + A_2 \times {\mathrm{exp}}( - (x - x_0)/{{t}}_2)$$

and the fluorescence lifetime is calculated as 4.56 ns by the formula below:

3 $${{t}} = (A_1{{t}}_1^2 + A_2{{t}}_2^2)/(A_1{{t}}_1 + A_2{{t}}_2) = 4.56\;{\mathrm{ns}}.$$

## Supplementary information

Supplementary Information

Peer Review File

Description of Additional Supplementary Files

Supplementary Movie 1

Supplementary Movie 2

Supplementary Movie 3

Supplementary Movie 4

## Data Availability

Data are available from the corresponding author upon reasonable request.
