# Supplementary material for: Hydrophobic carbon dots with blue dispersed emission and red aggregation-induced emission
Source: Nat Commun. 2019 Apr 17;10:1789. doi: 10.1038/s41467-019-09830-6 (PMC6470214; doi:10.1038/s41467-019-09830-6)
Supplement: Supplementary file 1 — Supplementary Information [file 41467_2019_9830_MOESM1_ESM.pdf]

Supplementary information to:

Hydrophobic carbon dots with blue dispersed emission and red aggregation-induced emission

Haiyao Yang et al.

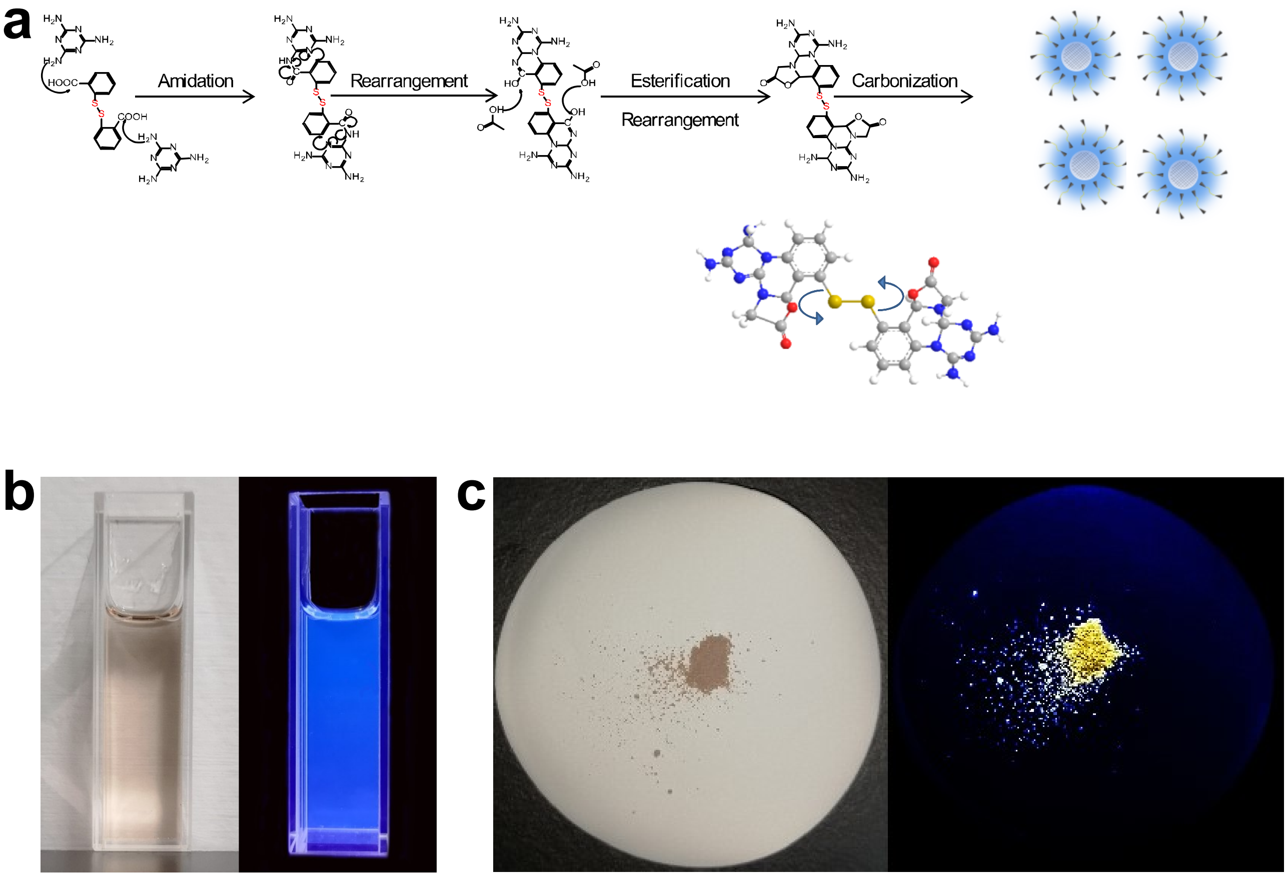

Supplementary Fig. 1 Display of H-CDs' formation, structure and appearance in different state. (a) A proposal mechanism of chemical reactions happened during the solvothermal treatment. Photographs of (b) PA-CDs solution and (c) powders under sunlight (left) and 365 nm UV (right).

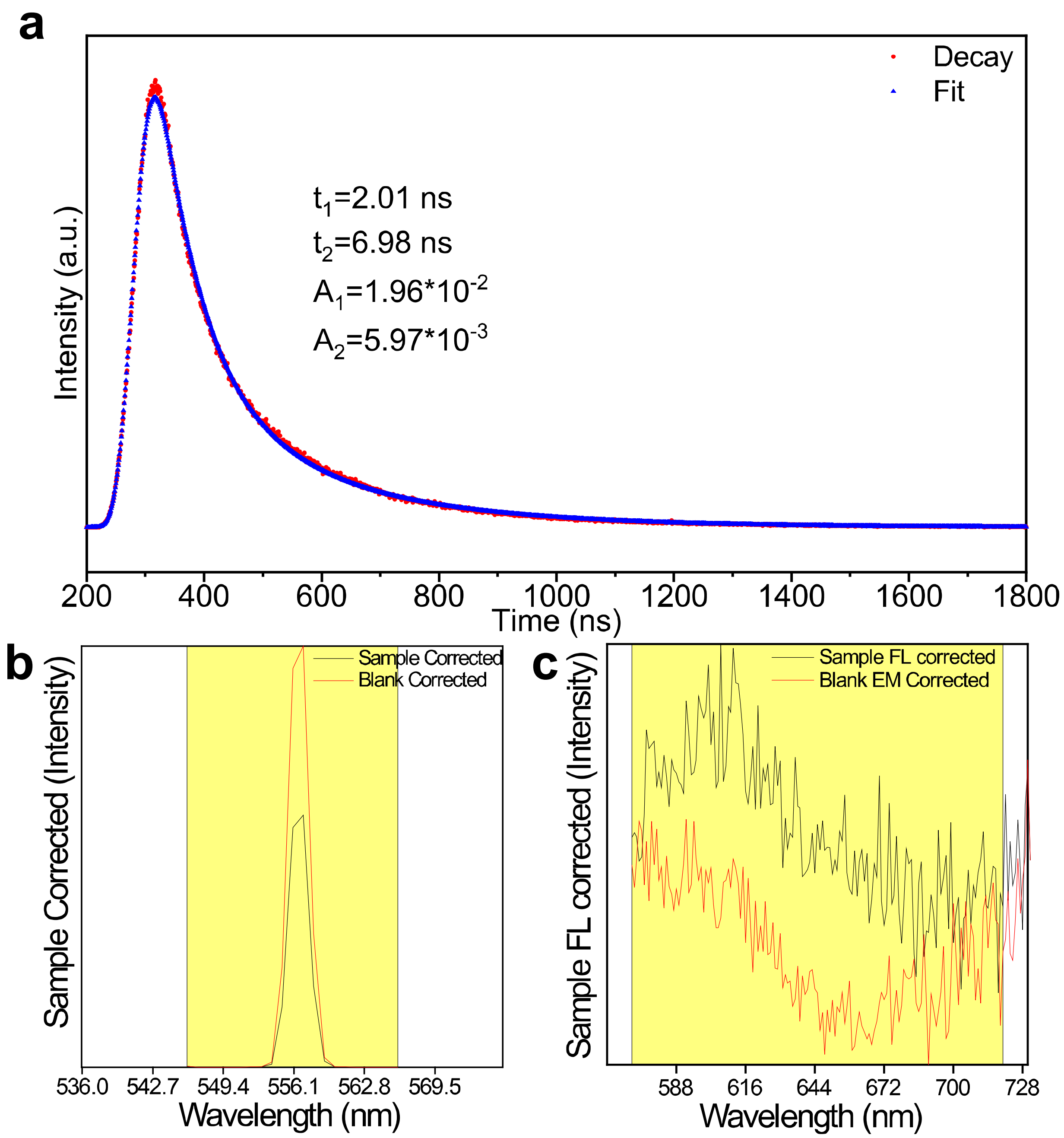

Supplementary Fig. 2 Fluorescence Lifetime and quantum yield. Fluorescence lifetime (a), photon absorption (b) and emission (c) of H-CDs powders.

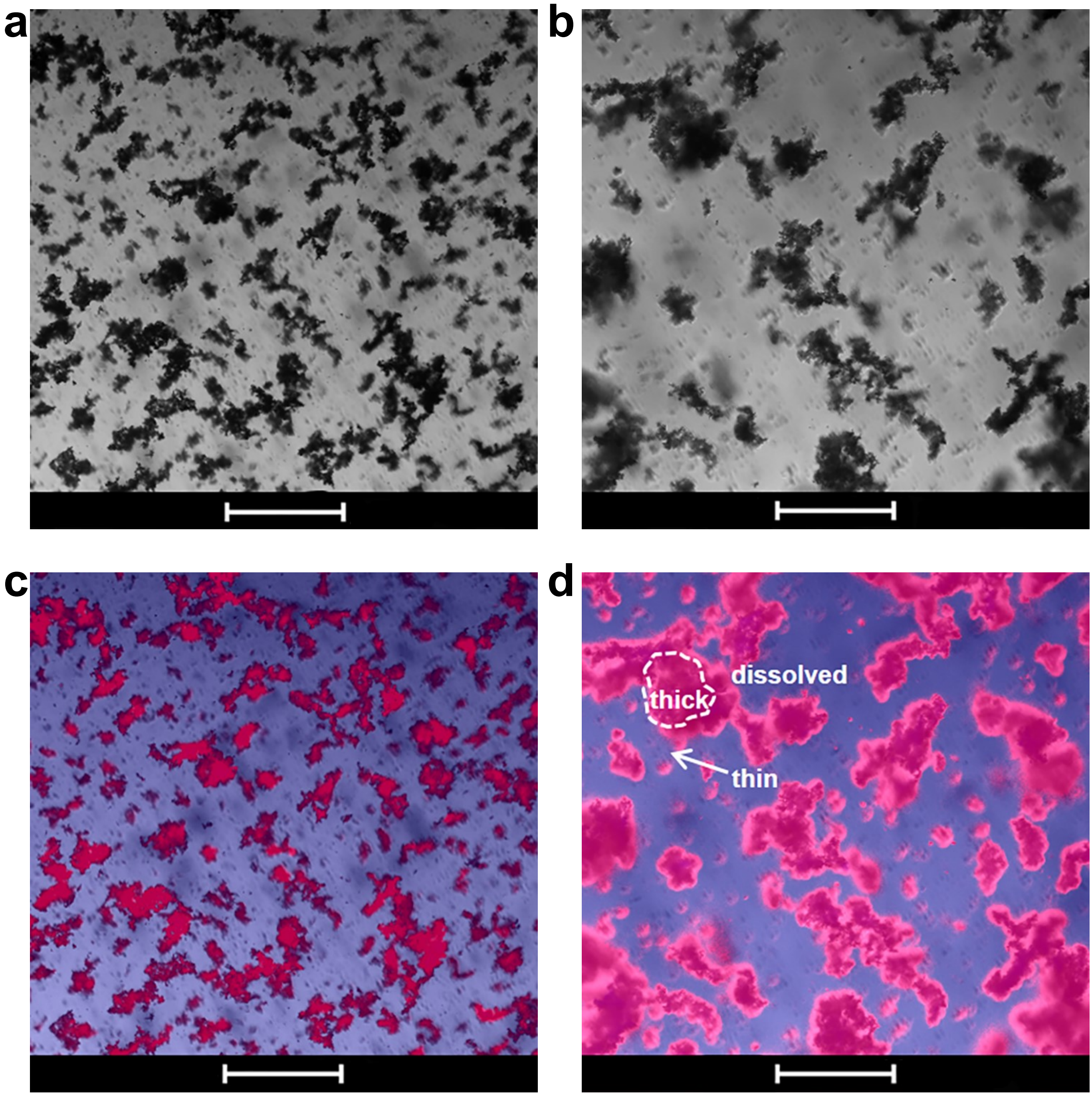

Supplementary Fig. 3 Bright field and fluorescence microscopy of H-CDs in different state. Bright field (a, b) and fluorescence microscopy (c, d) images of excessive H-CDs powders in ethanol. Scale bars: 200 μm (a, c), 100 μm (b, d).

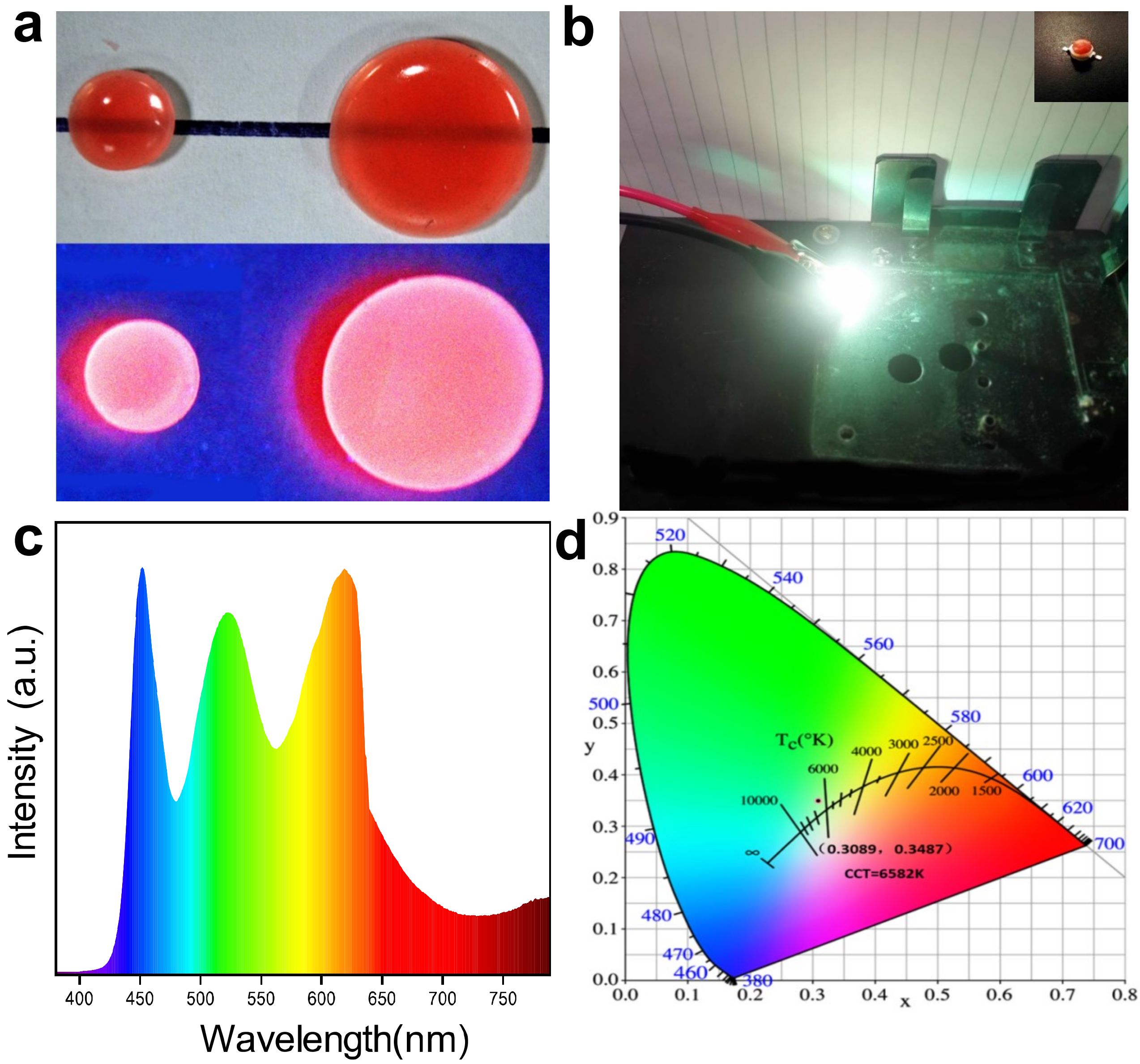

Supplementary Fig. 4 LED application of H-CDs glass. (a) Photographs of fluorescent organic glasses under sunlight (above) and 365nm UV (down). (b) Fluorescent images of WLED operated at 3.5 V (Inset: Optical images of the WLED). (c) CIE chromaticity coordinate and (d) PL emission spectra of the WLED.

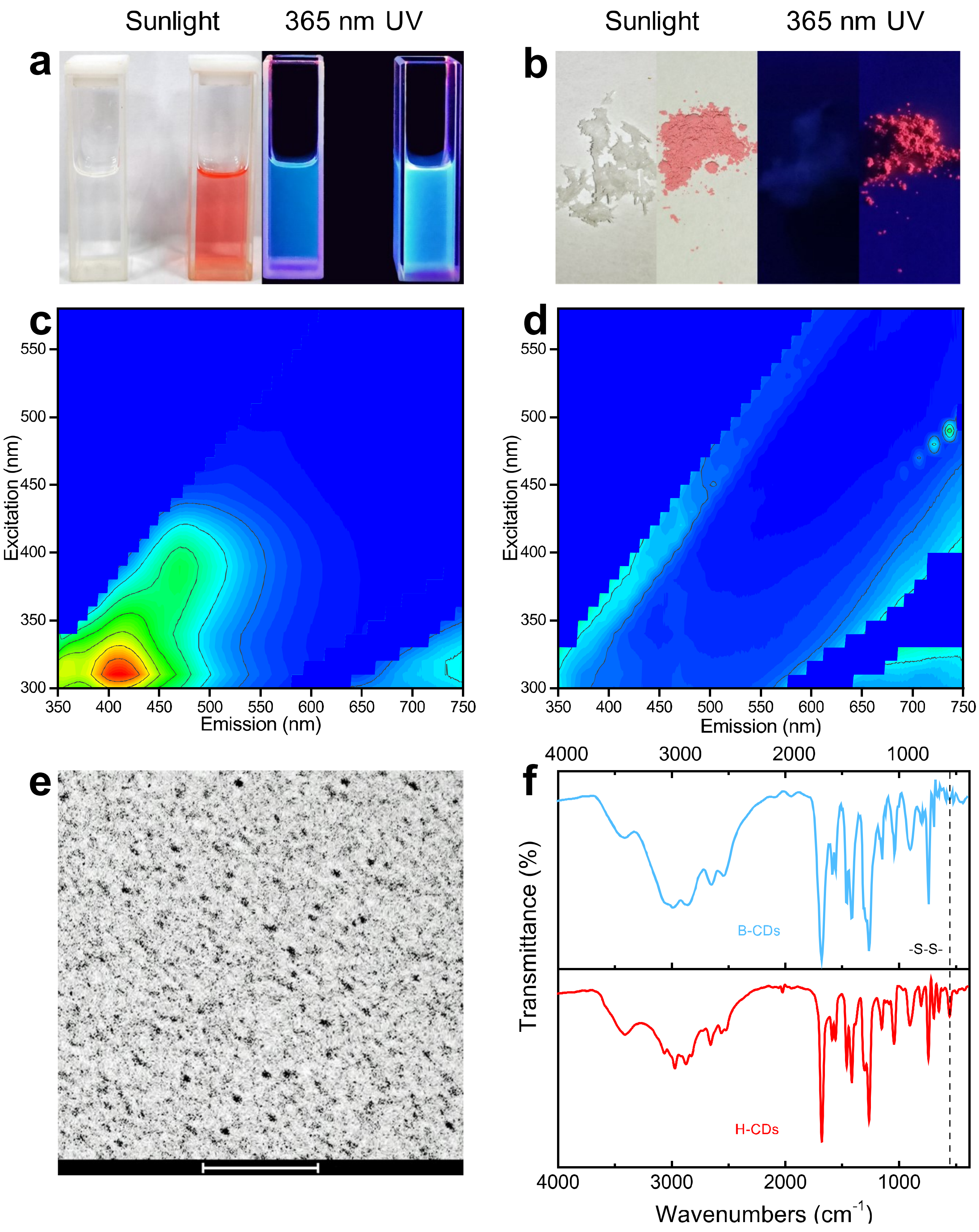

Supplementary Fig. 5 B-CDs' composition, morphology, fluorescence and appearance compared with H-CDs. (a) Photos of B-CDs (left) and H-CDs (right) ethanol solution under sunlight and 365 nm UV. (b) Photos of B-CDs (left) and H-CDs (right) powders under sunlight and 365 nm UV. PL mapping spectra of (c) B-CDs as-prepared solution, (d) B-CDs. (e) TEM of B-CDs powders dissolved in water. (f) FT-IR spectra of H-CDs and B-CDs. Scale bars: 100 nm (e).

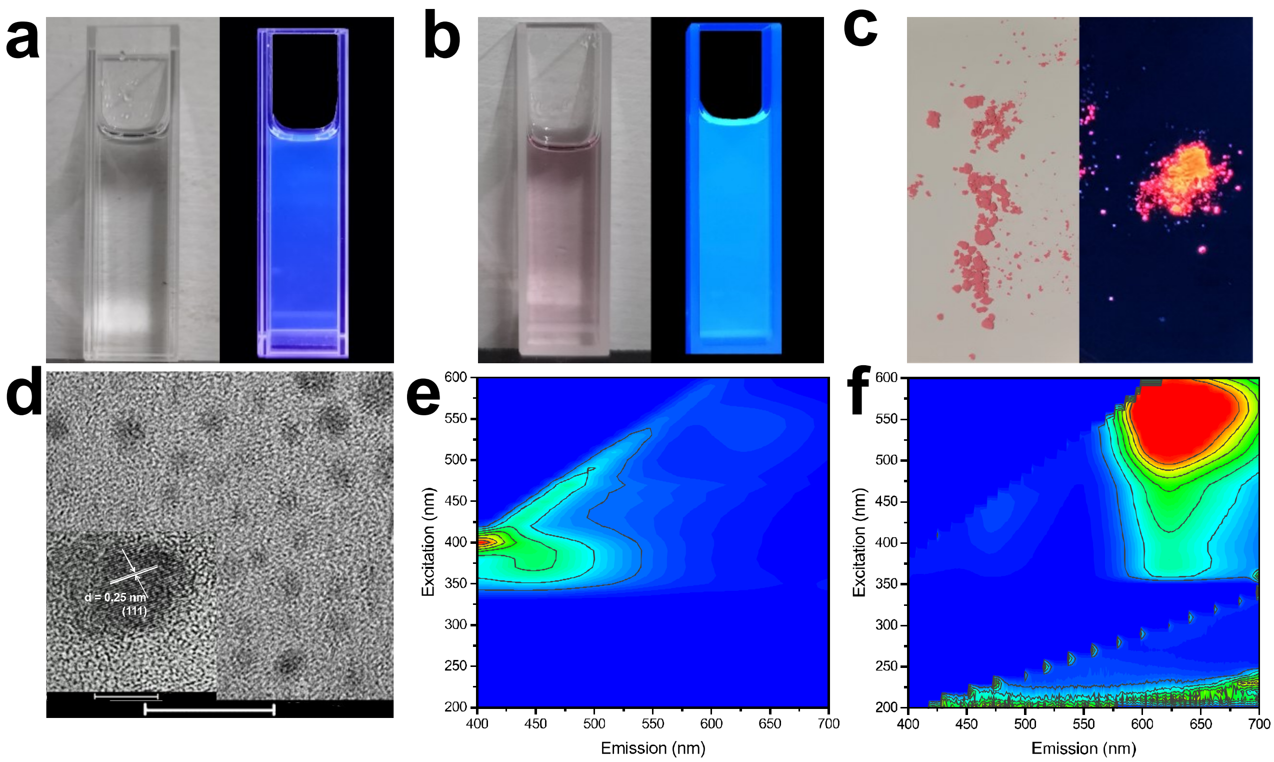

Supplementary Fig. 6 P-CDs compared with H-CDs. (a) Photos of P-CDs intermediate's water solution under sunlight(left) and 365 nm UV irradiation(right). (b) Photos of P-CDs as-prepared solution under sunlight (left) and 365 nm UV irradiation (right). (c) Photos of P-CDs powders under sunlight (left) and 365 nm UV irradiation (right). (d) TEM image of P-CDs intermediate (inset: HR-TEM image). PL mapping spectra of (e) P-CDs as-prepared solution and (f) powders. Scale bars: 100 nm (d) and 10 nm (d-inset).

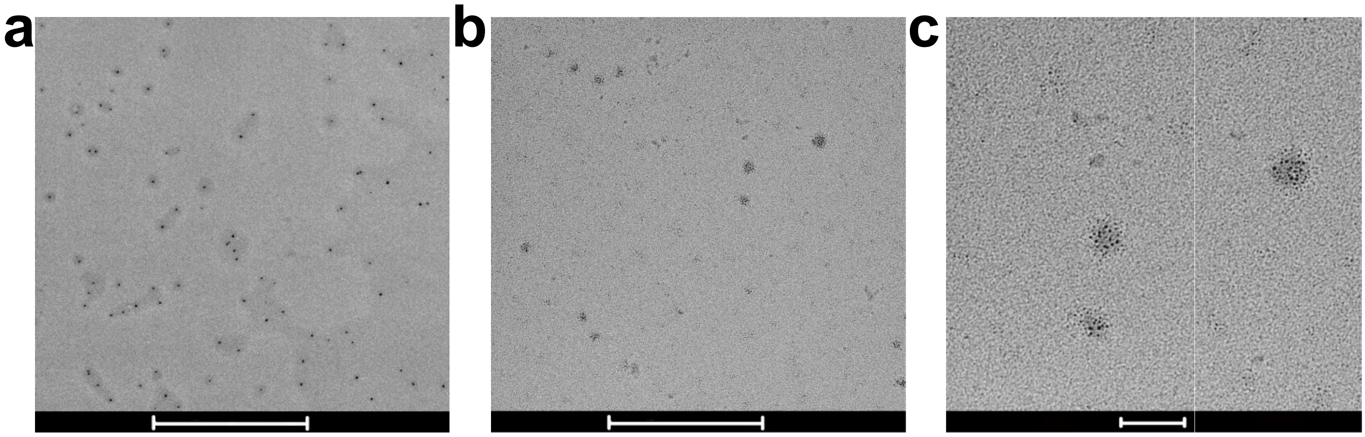

Supplementary Fig. 7 (a, b, c) TEM images of water after-treated H-CDs ethanol solution. Scale bars: 2 μm (a), 500 nm (b) and 100 nm (c).

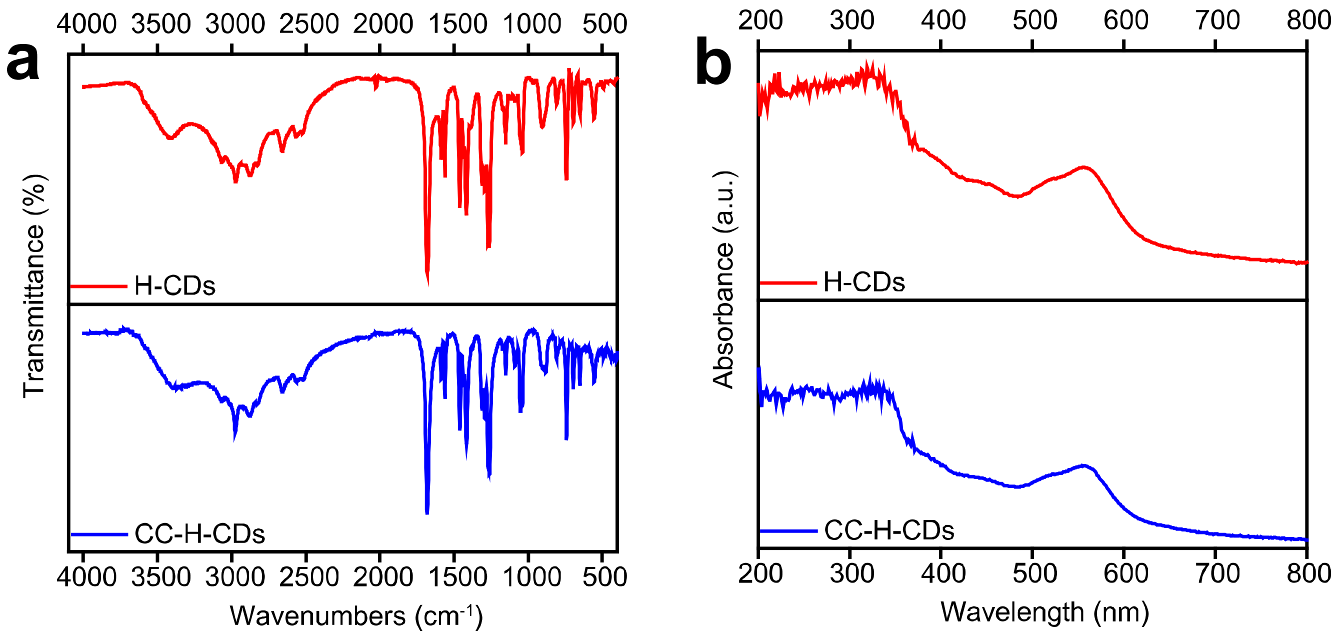

Supplementary Fig. 8 Composition of H-CDs and CC-H-CDs. (a) FT-IR and (b) UV-vis spectra of H-CDs and CC-H-CDs.
