# Supplementary material for: Hydrophobic carbon dots with blue dispersed emission and red aggregation-induced emission
Source: Nat Commun. 2019 Apr 17;10:1789. doi: 10.1038/s41467-019-09830-6 (PMC6470214; doi:10.1038/s41467-019-09830-6)
Supplement: Supplementary file 3 — Description of Additional Supplementary Files [file 41467_2019_9830_MOESM3_ESM.pdf]

Description of Additional Supplementary Files

File Name: Supplementary Movie 1
Description: Appearance and fluorescence of H-CD ethanol solution before and after injecting water.

File Name: Supplementary Movie 2
Description: H-CD ethanol solution under microscope.

File Name: Supplementary Movie 3
Description: H-CD ethanol solution after adding water under microscope.

File Name: Supplementary Movie 4
Description: Demonstration of H-CD ink written on filter paper with a series treatment.
