# Supplementary material for: Hydrophobic carbon dots with blue dispersed emission and red aggregation-induced emission
Source: Nat Commun. 2019 Apr 17;10:1789. doi: 10.1038/s41467-019-09830-6 (PMC6470214; doi:10.1038/s41467-019-09830-6)
Supplement: Supplementary file 2 — Peer Review File [file 41467_2019_9830_MOESM2_ESM.pdf]

Reviewers' comments:

Reviewer #1 (Remarks to the Author):

In this manuscript, the authors reported hydrophobic carbon dots (H-CDs) with reversible two-switch-mode luminescence, i.e., blue dissolved fluorescence and red solid-state fluorescence (SSF). Moreover, they proposed that the formation of H-CDs cluster induces turning off of blue emission as aggregation-caused-quenching (ACQ) caused by π-π stacking interaction of the carbonized cores, and turning on of red SSF due to restriction of the surfaces' intramolecular rotation (RIR) around disulfide bonds, which conforms to the aggregation-induced-emission (AIE) phenomenon. Finally, the anti-fake fluorescence ink for advanced anti-counterfeiting and dual encryption has been fabricated based on this two-switch-mode luminescence of H-CDs.

The material and phenomena of this study are interesting, but many flaws still existed:

1) There are numerous typo, spelling and grammar errors in this manuscript, please check and correct the manuscript carefully again.

2) The purification of the H-CDs is described in this manuscript as following: "After the solvothermal treatment, the as-prepared H-CDs solution was added into 1 L boiled water to form H-CDs powders and wash out residual raw materials, finally purified H-CDs powders were achieved through vacuum filtration." Clearly, such a procedure is not sufficient for purification of a material; column chromatography is suggested to be applied.

3) UV-Vis absorption and FL excitation wavelength regions should be extended in Figure 2a and 2b, so as one can better compare the H-CDs in solution and powder forms; more excitation wavelengths should be used to observe the emission property of the H-CDs in Figure 2c and 2d.

4) In page 8 (line 187-188), the authors stated that "as-prepared H-CDs solution (Fig. 2c) exhibit excitation-independent PL feature of most CDs from prior works", but this is clearly not the truth, because in Figure 2c, an excitation dependent PL feature was observed!

5) In page 8 (line 192), the authors claimed that "The fluorescence lifetime of H-CDs is measured as 970 ns (Fig. S2a)", this is a clear mistake again!

6) In page 8 (line 189-191), the authors stated that "The calculated energy level transitions (Fig. 2e) of H-CDs' carbonized cores and symmetrical heterocyclic surfaces can be according to their absorptive and emissive properties". Herein, the authors should give more details about how to calculate the energy level transitions of the H-CDs, and corresponding discussion should also be provided!

7) From the data of Figure 4d and 4e, the H-CDs seem exhibit two size distributions that indicating their dispersion and aggregation states, respectively. So Figure 4f is misleading.

8) In page 3 (line 99-101), the authors stated that "It is well to be reminded that acetic acid is irreplaceable for the successful preparation of H-CDs (all the other solvents including water, ethanol, acetone and the like do not work)." However, is it really that the acetic acid cannot be replaced? How about other organic acid such as formic acid, propionic acid, oxalic acid and so on? In addition, the role of acetic acid should be further discussed for the synthesis of H-CDs.

9) To further verify the fluorescence mechanism of H-CDs, the authors carried out a control-experiment, which displaced dithiosalicylic acid (DTSA) with benzoate to remove the effect of disulfide-bonds. Such a control experiment is not sufficient. I suggest the authors to take a post-modification method to synthesize dithiosalicylic acid-modified CDs, and then compare their PL property with H-CDs.

Reviewer #2 (Remarks to the Author):

The authors reported H-CDs which shows blue emission in the ethanol or acetic acid solution and red emission in the solid state due to the formation of aggregation. The blue emission and red emission can reversibly change in the dissolution and aggregates state. However, it is not fully understood for this phenomena.

1. The English need to be improved, it is really hard to understand. The Figure order needs to be checked.

2. The formation of aggregation needs to be further investigated, such as, what kind aggregation (H or J)? Does it belong to p-p stacking?

3. TEM images belong to localized characterization, provide spectral evidence to prove the aggregation formation.

4. Normally, aggregation induced quench can be found in the fluorescence. Why does this system exhibit AIE phenomenon? Provide enough evidence.

5. What is the PL excitation spectrum from 200 to 600? Why can H-CDs be excited 254 nm, not 365 nm?

6. Provide a detailed explanation of Figure 5i.

7. How about PL QY of H-CDs with excitation of 570 nm?

8. How to decide the PL lifetime 970 ns?

## Reply to reviewer 1

We sincerely thank you for your comments and suggestions which have helped us greatly improved the quality of this paper. In the following, we give a point-by-point reply to your comments. Your comments are shown in ***italics*** and are then followed by our responses.

*Comments:*

*In this manuscript, the authors reported hydrophobic carbon dots (H-CDs) with reversible two-switch-mode luminescence, i.e., blue dissolved fluorescence and red solid-state fluorescence (SSF). Moreover, they proposed that the formation of H-CDs cluster induces turning off of blue emission as aggregation-caused-quenching (ACQ) caused by π-π stacking interaction of the carbonized cores, and turning on of red SSF due to restriction of the surfaces' intramolecular rotation (RIR) around disulfide bonds, which conforms to the aggregation-induced-emission (AIE) phenomenon. Finally, the anti-fake fluorescence ink for advanced anti-counterfeiting and dual encryption has been fabricated based on this two-switch-mode luminescence of H-CDs.The material and phenomena of this study are interesting, but many flaws still existed:*

***Comment 1**: There are numerous typo, spelling and grammar errors in this manuscript, please check and correct the manuscript carefully again.*

**Response:** Thank you for this comment. We have cautiously checked and corrected the manuscript again. We also used a professional editing service provided by ***Nature Research Editing Service*** to improve the English.

***Comment 2**: The purification of the H-CDs is described in this manuscript as following: "After the solvothermal treatment, the as-prepared H-CDs solution was added into 1 L boiled water to form H-CDs powders and wash out residual raw materials, finally purified H-CDs powders were achieved through vacuum filtration." Clearly, such a procedure is not sufficient for purification of a material; column chromatography is suggested to be applied.*

**Response:** This is a good question. The raw materials and solvent of the H-CDs are both soluble in hot water, while the H-CDs are hydrophobic even in boiled water. So we took the foresaid method to fabricate H-CDs powders and purify them at the same time in a convenient way.

However, the dependability of this purification was not verified. Thus, we applied column chromatography to purify the as-prepared H-CDs solution, according to the reviewer's suggestion. H-CDs powders were fabricated through water washing, centrifugation and vacuum drying. To differentiate, the H-CDs powders obtained from this approach are named as CC-H-CDs. The purification effect of boiled water and column chromatography were compared by FT-IR and UV-vis spectra (Supplementary Fig. 8) of H-CDs and CC-H-CDs powders. As the component of H-CDs powders achieved from column chromatography and our boiled water wash method is accordant, we confirmed our purification reliable. This information has been added to the text and Supplementary information as described below:

Page 22-23: To confirm the reliability of this water-wash method, we applied column chromatography to purify the H-CD solution for comparison, and the H-CDs obtained from this approach are named CC-H-CDs. FT-IR and UV-vis spectra of H-CDs and CC-H-CD powders in Supplementary Fig. 8 suggest the components are approximately identical. Therefore, the

water-wash method is considered as reliable as column chromatography.

***Comment 3**: UV-Vis absorption and FL excitation wavelength regions should be extended in Figure 2a and 2b, so as one can better compare the H-CDs in solution and powder forms; more excitation wavelengths should be used to observe the emission property of the H-CDs in Figure 2c and 2d.*

**Response:** Well spotted, we have revised the manuscript according to your comment. Please see page 10 (line 171-177).

***Comment 4**: In page 8 (line 187-188), the authors stated that "as-prepared H-CDs solution (Fig. 2c) exhibit excitation-independent PL feature of most CDs from prior works", but this is clearly not the truth, because in Figure 2c, an excitation dependent PL feature was observed!*

**Response:** Thank you for this comment. We have corrected this typo mistake. Please see page 11 (line 189).

***Comment 5**: In page 8 (line 192), the authors claimed that "The fluorescence lifetime of H-CDs is measured as 970 ns (Supplementary Fig. 2a)", this is a clear mistake again!*

**Response:** We have recalculated the fluorescence lifetime and corrected this typo mistake, and the details of this computational process have been added to the method section, please see page 24, line 444-447.

***Comment 6**: In page 8 (line 189-191), the authors stated that "The calculated energy level transitions (Fig. 2e) of H-CDs' carbonized cores and symmetrical heterocyclic surfaces can be according to their absorptive and emissive properties". Herein, the authors should give more details about how to calculate the energy level transitions of the H-CDs, and corresponding discussion should also be provided!*

**Response:** This is a very important point. We have provided computational process and corresponding discussion of energy level in the method section, please see page 24, line 437-444.

***Comment 7**: From the data of Figure 4d and 4e, the H-CDs seem exhibit two size distributions that indicating their dispersion and aggregation states, respectively. So Figure 4f is misleading.*

**Response:** This is a good question. We have replaced this size distribution diagram, and the aggregates it refers to have been marked to avoid misunderstanding. Please see page 14-15 (line 247-253).

***Comment 8**: In page 3 (line 99-101), the authors stated that "It is well to be reminded that acetic acid is irreplaceable for the successful preparation of H-CDs (all the other solvents including water, ethanol, acetone and the like do not work)." However, is it really that the acetic acid cannot be replaced? How about other organic acid such as formic acid, propionic acid, oxalic acid and so on? In addition, the role of acetic acid should be further discussed for the synthesis of H-CDs.*

**Response:** Thank you for this comment, we were meant to emphasize the importance of acetic acid in H-CDs' formation by using the word "irreplaceable". However, this expression is not accurate, we applied a series of control-experiment which replaced acetic acid by formic acid,

propionic acid and saturated aqueous solution of oxalic acid. When propionic acid is applied, the product shows similar PL property with H-CDs: blue emission in dispersion and AIE in powder state. Therefore, acetic is actually replaceable. However, propionic acid is much more expensive and poisonous than acetic acid, and the fluorescence of PA-CDs powders is just yellow other than the red AIE of H-CDs. We have revised the manuscript. Please see page 4-5 (line 96-102).

***Comment 9****: To further verify the fluorescence mechanism of H-CDs, the authors carried out a control-experiment, which displaced dithiosalicylic acid (DTSA) with benzoate to remove the effect of disulfide-bonds. Such a control experiment is not sufficient. I suggest the authors to take a post-modification method to synthesize dithiosalicylic acid-modified CDs, and then compare their PL property with H-CDs.*

**Response:** This is a very helpful advice. We have carried out a post-modification method (the product of this method is named as P-CDs) and revised the manuscript. Firstly, MA is dissolved into acetic acid and proceeded with a solvothermal pre-treatment. P-CDs' intermediate is water-soluble and displays blue fluorescence (Supplementary Fig. 6a). Supplementary Fig. 6d displays the TEM image of the intermediate product and the inset indicate that a carbonized dot structure with 0.25 nm lattice spacing (111 lattice plane of carbon), which can further verify the blue emission of H-CDs comes from its carbonized core. P-CDs were then fabricated by mixing DTSA with aforementioned intermediate and acetic acid after a post-solvothermal processing. As shown in Supplementary Fig. 6b, c, e, f, P-CDs exhibit same hydrophobicity and PL property with H-CDs, which confirms the root of H-CDs' hydrophobicity and red AIE is the DTSA modified surface. Please see page 15 (line 254-275).

## Reply to reviewer 2

We are grateful for your comments, which have motivated us to improve the manuscript. In what follows, your comments are shown in ***italics*** and are then followed by our responses.

***Comments:*** *The authors reported H-CDs which shows blue emission in the ethanol or acetic acid solution and red emission in the solid state due to the formation of aggregation. The blue emission and red emission can reversibly change in the dissolution and aggregates state. However, it is not fully understood for this phenomena.*

***Comment 1****: The English need to be improved, it is really hard to understand. The Figure order needs to be checked.*

**Response:** We appreciate your comment. The manuscript has been cautiously checked and corrected. We also used a professional editing service provided by ***Nature Research Editing Service*** to improve the English.

***Comment 2****: The formation of aggregation needs to be further investigated, such as, what kind aggregation (H or J)? Does it belong to p-p stacking?*

**Response:** This is a good question. To further confirm the formation of aggregation. We applied a post-modification method to synthesize dithiosalicylic acid-modified CDs, updated the video of H-CDs ethanol solution's aggregation process by adding water under 365nm UV irradiation, added two videos taken by fluorescence microscope before and after adding water (Supplementary Vid. 1, 1a, 1b), took different sample preparation of TEM imaging (Supplementary Fig. 7). Which have helped us to further confirm the carbonized core with hydrophobic surface structure of H-CDs, and the fluorescent character resulted from this structure.

The Conjugated system of organic molecules and nanoparticles can form two distinct types of $\pi-\pi$ aggregate, a sandwich-type arrangement (H-aggregates) and a head-to-tail arrangement (J-aggregates) (Ref.42). To validate the form of carbon dots' or dye molecules' form of aggregation, most researchers applied absorption spectra (Ref. 43, 44, 45, 46). Following the commendation of these studies on the aggregation formation of carbon dots, we applied absorption spectra to investigate the formation of aggregation. With the injection of water, the absorbance of H-CDs solution at 360 nm keeps decreasing, while a red-shifted absorbance at 559 nm appears and keeps increasing. Which provides strong evidence for the presence of $\pi-\pi$ stacking in H-CDs. According to the research on the formation of aggregation of carbon dots carried out by Li et al (Ref.42) and Chen et al (Ref.43), based on molecular exciton coupling theory, this spectral red-shift indicates that the H-CDs form J-aggregates, with a head-to-tail arrangement. Summarily, we conclude that the formation of aggregation is induced by synergistic effect of surfaces' hydrophobicity and $\pi-\pi$ stacking. When the H-CD monomers contact water, the hydrophobicity of their surfaces make them approach each other, then the conjugated systems conduct $\pi$-$\pi$ stacking of the surface to overlay each other and finally take the shape of J-aggregates. We have revised the text to further discuss the formation of aggregation, please see page 12-13, line 210-232.

***Comment 3****: TEM images belong to localized characterization, provide spectral evidence to prove the aggregation formation.*

**Response:** As addressed in answer to comment 2, we have provided absorption spectra as an

evidence for the aggregation formation. What's more, the fluorescence spectra (Fig. 3b, c) can also prove the taking place of aggregation during the water injection, as the appearance and increase of a red-shifted emission at 620 nm.

***Comment 4**: Normally, aggregation induced quench can be found in the fluorescence. Why does this system exhibit AIE phenomenon? Provide enough evidence.*

**Response:** As you mentioned，normally carbon dots suffer fluorescence due to the π−π stacking of fluorophore after their aggregation. However, Tang' group (Ref. 15) discovered a series of organic fluorescent materials and found that luminogen aggregation played a destructive role in the light-emitting process. In their study, a series of symmetrical molecules were found to be nonluminescent in the dissolved state, but emissive in the aggregated state. The term "aggregation-induced emission" (AIE) was coined for this novel phenomenon, as the nonluminescent symmetrical molecules were induced to emit via aggregate formation. after that, amount of molecules with AIE property have been discovered. Among these AIE molecules, some occupy similar structure: conjugated systems around an axis of symmetry (Ref. 15-18). The authors approved a theory, that in dispersed state AIE molecules will consume the absorbed energy due to their rotation about the symmetric axis, once the molecules turn into aggregation the restriction of intramolecular rotation enable them to apply the energy to glow.

In accordance with the structure of H-CDs' raw material (DTSA) which contains a disulfide bond, we have proposed a mechanism of chemical reactions happened during the solvothermal treatment (Supplementary Fig. 1a). Via this reaction mechanism, symmetric structure around H-CDs' surfaces like the AIE molecules abovementioned. Structural characterizations such as FT-IR, XPS, Raman spectra, XRD and NMR are accordant to the conclusion that the H-CDs are carbonized cores covered with abovementioned amorphous surfaces. Moreover, through two controlled experiments (page 15, line 254-275), we have further confirmed the abovementioned surface is the origin of H-CDs' red AIE and hydrophobicity, and the disulfide bond in it is essential as an axis of symmetry. While the blue emission of dispersed H-CDs is proved to stem from the carbonized cores. We hence consider the aggregation of H-CDs solution into powders is along with the ACQ of their cores and AIE of surfaces. Please see page 15-16 (line 254-296) and Supplementary Vid. 1, 1a, 1b.

***Comment 5**: What is the PL excitation spectrum from 200 to 600? Why can H-CDs be excited 254 nm, not 365 nm?*

**Response:** UV-Vis absorption and PL excitation wavelength regions have extended in Figure 2a and 2b.

Due to the excitation spectra, both H-CDs solution and powders can be excited 365 nm, while H-CDs solution cannot be excited 254 nm but H-CDs powders can. By comparing the absorption of H-CDs solution and powders (page 10, Fig. 2a, b), we have learnt that at 254 nm, H-CDs powders exhibit strong absorption, while H-CDs solution shows extremely low absorption. We consider that is why H-CDs solution cannot be excited 254 nm.

***Comment 6**: Provide a detailed explanation of Figure 5i.*

**Response:** The square frames in Figure 5i represent the filter paper; the wavy lines represent the

paper's fibers. Blue dots represent the H-CD monomers dispersed in the filter paper due to the restriction of the paper's fibers. As mentioned above, H-CD monomers cannot be excited at 254 nm but can be excited at 365 nm. When water is introduced, some of the H-CD monomers aggregate and surface. Furthermore, the other monomers remain joint to the fibers. Therefore, under 365 nm irradiation, both the monomers in the filter paper and the aggregates on surface can be excited to glow blue and red emission, which display as a hybrid pink fluorescence. While under 254 nm irradiation, the monomers are not excited further, resulting in the red emission only. Once ethanol is applied, the aggregates will dissolve into the filter paper as monomers again; therefore, this process is reversible. Please see page 17-18 (line 310-331)

***Comment 7**: How about PL QY of H-CDs with excitation of 570 nm?*

**Response:** According to the excitation and absorption curves of H-CDs powders in Fig. 2b, when the excitation exchange from 559 nm to 570 nm, the intensity of these two curves decrease together, and the PL QY is correlated to absorption intensity divide excitation intensity. Therefore, the PL QY of H-CDs with excitation of 570 nm can be predicted similar to it of 559 nm. Actually, the PL QY of H-CDs with excitation of 570 nm is measured as 5.56%, which is slightly lower than the best excitation 559nm:

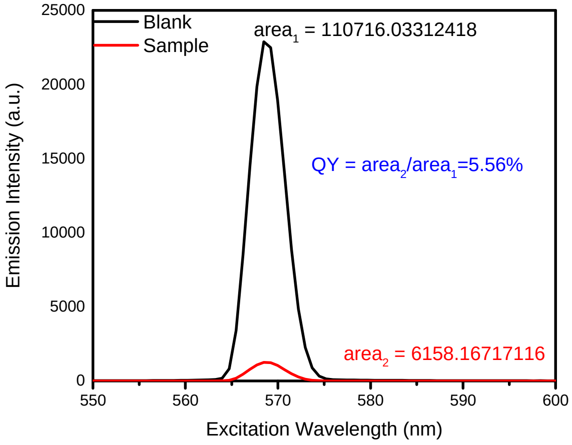


**Extra Figure 1: photon absorption (b) and emission (c) of H-CDs powders with excitation of 570 nm.**

***Comment 8**: How to decide the PL lifetime 970 ns?*

**Response:** We are sorry that the PL lifetime 970 ns is a typo mistake, the fluorescence lifetime has been recalculated and this typo mistake has been corrected:

Page 11: The fluorescence decay curve and double-exponential fitting result of it are shown in Supplementary Fig. 2a, the fitting function is as the formula below

$$y = y_0 + A_1 * \exp(-(x - x_0)/t_1) + A_2 * \exp(-(x - x_0)/t_2) \qquad (2)$$

, the fluorescence lifetime is calculated as 4.56 ns (Supplementary Fig. 2a). The details of this computational process have been added to the method section, please see page 24, line 444-447.

REVIEWERS' COMMENTS:

Reviewer #1 (Remarks to the Author):

The authors had replied my concerns and modified the manuscript properly, so I think this manuscript can be accepted at this stage.

Reviewer #2 (Remarks to the Author):

The author have addressed what I am concerned
